# Simulation Experiment Research of Mine Roadway Simulating Test Device with Adjustable Wind Velocity and Temperature and Humidity

**DOI:** 10.3390/ijerph20054057

**Published:** 2023-02-24

**Authors:** Lindong Liu, Cuifeng Du, Yuan Wang, Jianwu Chen, Bin Yang, Weibo Jin

**Affiliations:** 1 School of Civil and Resources Engineering, University of Science and Technology Beijing, Beijing 100083, China; 2 Key Laboratory of Ministry of Education for Efficient Mining and Safety of Metal Mine, Beijing 100083, China; 3 NHC Key Laboratory for Engineering Control of Dust Hazard, Beijing 100083, China; 4 Institute of Occupational Health, China Academy of Safety Science and Technology, Beijing 100012, China

**Keywords:** wind velocity, temperature and humidity, mine roadway, non-uniformity

## Abstract

The design and development process of wind velocity sensors for mining has been a challenging task due to the complexity of a large number of field tests. To resolve this problem, this study aimed to provide a comprehensive test device for the design and development of high-precision wind velocities sensor for mining. Through a combination of experiments and computational fluid dynamics (CFD), a device that can simulate the mine roadway environment was developed. The device can control the temperature, humidity, and wind velocity parameters to fully replicate the mine roadway environment. It gives designers and developers of high-precision wind velocity sensors for mining a rational and scientific testing environment. In order to quantitatively define the uniformity of air flow in the mine highway section, the research introduced the non-uniformity determination method. The approach was expanded to assess the cross-sectional uniformity of temperature and humidity. The wind velocity within the machine can increase to 8.5 m/s by selecting the right kind of fan. The minimum wind velocity non-uniformity at this moment is 2.30%. The device’s internal temperature can be raised to 38.23 °C and its humidity level can be increased to 95.09% by carefully crafting the rectifier orifice plate’s structure. At this time, the lowest temperature non-uniformity is 2.22%, and the lowest humidity non-uniformity is 2.40%. The device’s average wind velocity is 4.37 m/s, its average temperature is 37.7 °C, as well as its average humidity is 95%, per the emulate results. The device’s non-uniformity in wind velocity, temperature, and humidity is 2.89%, 1.34%, and 2.23%, respectively. It is capable of simulating the mine roadway environment in its entirety.

## 1. Introduction

The ventilation system is the fundamental system to ensure the safety of mine production. According to statistics, many accidents are caused by ventilation system breakdowns that seem to go unnoticed in time [1]. It is crucial to accurately, and in real time, monitor the operation of the mine ventilation system [2]. The mainstream method for achieving real-time monitoring of mine ventilation at the moment is to utilize an online monitoring system for mine ventilation [3]. The ventilation monitoring system’s initial node of data collection is the mine wind velocity sensor. The mine roadway’s high temperature and high humidity environment may easily have an impact on data gathering, leading to poor accuracy wind velocity data [4,5]. Therefore, the design and development of high-precision wind velocity sensors for mining must take into account adapting to the subterranean high temperature and high-humidity environment. This will effectively enhance the accuracy of the ventilation monitoring system. There must be several field tests conducted on the mine wind velocity sensor design and development. Downhole measurements, however, are time- and money-consuming. More significantly, a number of the highly accurate instruments used in the research have trouble operating correctly in the hot, humid downhole environment. Thus, hence, a scientific and reasonable test device for the design and development of mine wind velocity sensor can be provided by the design and development of mine roadway mimicking testing machine with adjustable wind velocity, temperature, and humidity. It is essential to increase the wind velocity in mine roadway monitoring accuracy and ensuring the safety of mine production.

Creating the mimicking test equipment is premised on mastering the mine roadway’s environmental parameters. The wind velocity, temperature, and humidity parameters of mine roadway air flow have already been tested and simulated by many scholars. They believe that the wind current in the mine roadway is basically distributed in parallel streamlines and that the wind velocity is distributed evenly in the same vertical section [6]. The tunnel environment, however, cannot be comprehensively be reflected by the research on wind velocity alone. Environment impact factors, including temperature and humidity, must also be considered. The work by Li developed a fully coupled model capable of simulating the non-isothermal flow inside the roadway and heat transfer in surrounding rock, together with moisture transfer driven by water evaporation [7]. The study by Peltier presented a fundamental investigation of the convection heat transfer phenomenon driven by airflows in underground tunnels, with reference to the development of the so-called thermal and velocity boundary layers [8]. By analyzing substantial quantities of temperature and humidity data from a coal mine, Su carried out a mathematical simulation of the thermal environment of a high-temperature coal mine roadway to obtain results [9]. Research demonstrates that if the impact of highway humidity is ignored, it is impossible to adequately represent the environmental parameters of a roadway. The air humidity also has an impact on the airflow temperature in the roadway [10,11]. Regarding mine ventilation in a tunnel with one mining face, the turbulent flow structure of fresh air discharged from the ventilation ducts are numerically studied using the ANSYS and flow streamlines, velocity vectors, eddy viscosity, and total pressure in the tunnel of a mine with ventilation ducts are presented [12]. As a result of flow parameter measurements performed in mine drifts utilizing the hot-wire anemometric system of multipoint simultaneous flow velocity and temperature measurements based on integrated measurement heads, a set of data describing the parameters of flow in real transverse sections was obtained. Results of these measurements were used as a basis for further three-dimensional numerical modelling of the flow field structure in mine drifts [13]. This paper used CFD modeling to simulate the airflow behavior in underground crosscut regions [14]. This study utilizes the processing of data from laser scanning of a mine gallery, with simultaneous multi-point measurements of the velocity field at selected gallery cross-sections, unique for mine conditions [15]. On an experimental and numerical sense, the present paper deals with a study on ventilation systems. The measurements taken in a real mine gallery with a hot-wire anemometry have been used to validate the numerical model [16].

Based on the findings of the aforementioned investigation combined with the environmental parameters of the mine roadway measured data, the wind velocity, temperature, and humidity coupling device model was designed and constructed for this investigation using the CFD approach. For a complete simulation of the tunnel environment, it can control the wind velocity, temperature, and humidity parameters simultaneously. In a bid to quantitatively determine the uniformity of wind flow in the mine roadway section, the study applies the non-uniformity determination method [17]. The approach was expanded to assess the cross-sectional uniformity of temperature and humidity. By choosing the proper type of fan, the rectifier orifice plate’s structure may be optimized. We designed and implemented a simulating test device for mine roadways with adjustable wind velocity, temperature, and humidity.

## 2. Materials and Methods

### 2.1. Model Establishment

#### 2.1.1. Physical Model

**Figure 1:**
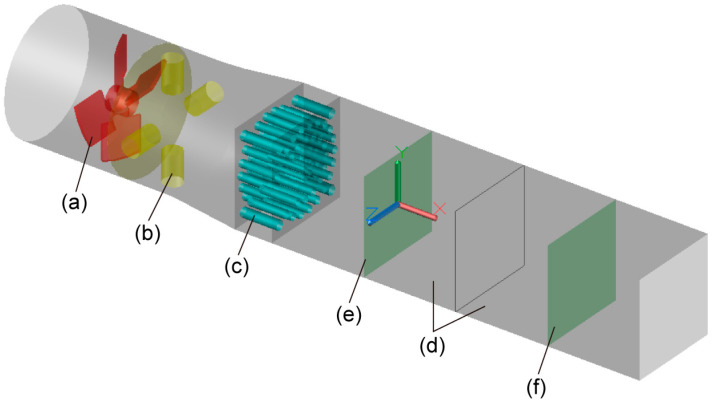
Test device structure description and monitoring surface position diagram. (**a**) fan; (**b**) humidifier pipe section (water mist inlet); (**c**) heating pipe section and rectifier orifice plate; (**d**) two sections of test pipe; (**e**) monitoring surface A; (**f**) monitoring surface B.

The device’s three-dimensional model was created using AutoCAD software and can be seen in Figure 1. It primarily consisted of a fan, a humidification pipe section, a heating pipe section, a rectifier orifice plate, and two test pipe sections. The fan used the same axial flow fan as mine tunnel air supply to the device in order to increase consistency between mine roadway air flow and device air flow. The humidification pipe part at the fan output was equipped with four symmetrically distributed water mist inlets, and the water mist flow was adjustable to manage the humidity. Electric heating tubes were used to warm the heating pipe segment. The collected results demonstrated that the mine tunnel section’s wind velocity, temperature, and humidity distribution were essentially uniform. Therefore, the installation of the rectifier orifice plate was required to accomplish the equal distribution of wind velocity, temperature, and humidity. The regularity of air flow may be significantly improved using rectifier orifice plates, according to studies [18,19]. Ingeniously, this invention suggests combining a heating tube with a rectifier orifice plate to simultaneously heat and correct the air flow.

The specific dimensions of the device were: fan diameter D_1_ = 620 mm, length L_1_ = 400 mm, impeller diameter D = 600 mm; the diameter of humidification pipe section D_2_ = 50 mm, length L_2_ = 100 mm; the length of the heating pipe section L_3_ = 200 mm, and the diameter was adjusted according to the rectifier effect of the rectifier orifice. The test pipe sections were square pipes with side length a = 500 mm and length L_4_ = 1000 mm. The origin of the device coordinate was located at the center of the test pipe section near the rectifier orifice plate. Figure 1 displays the location and motion of the coordinate axis. The middle cross profile of two test pipe portions that were located on device were chosen for data monitoring in order to track the parameters for wind velocity, temperature, and humidity. The monitoring surface was perpendicular to the *yOz* plane; monitoring surface A was located at *x* = 0, while monitoring surface B was located at *x* = 1000.

#### 2.1.2. Mathematical Model

Fluent, as a leading CFD software with a wide audience, was used to solve the model calculation of this study. The simulation process adopted double precision three-dimensional solver and *SST k-ω* Turbulence model, the internal flow field and heat transfer of the device model met the following equations:

The mass conservation equation is

(1) $$\frac{\partial u}{\partial x}+\frac{\partial v}{\partial y}+\frac{\partial w}{\partial z}=0,$$

The momentum conservation equation is

(2) $$\{\begin{array}{c} \frac{\partial \left(uu\right)}{\partial x}+\frac{\partial \left(uv\right)}{\partial y}+\frac{\partial \left(uw\right)}{\partial z}=\mu \left(\frac{\partial^{2}u}{\partial x^{2}}+\frac{\partial^{2}u}{\partial y^{2}}+\frac{\partial^{2}u}{\partial z^{2}}\right)-\frac{\partial p}{\partial x}\\ \frac{\partial \left(uv\right)}{\partial x}+\frac{\partial \left(vv\right)}{\partial y}+\frac{\partial \left(vw\right)}{\partial z}=\mu \left(\frac{\partial^{2}v}{\partial x^{2}}+\frac{\partial^{2}v}{\partial y^{2}}+\frac{\partial^{2}v}{\partial z^{2}}\right)-\frac{\partial p}{\partial y}\\ \frac{\partial \left(uw\right)}{\partial x}+\frac{\partial \left(vw\right)}{\partial y}+\frac{\partial \left(ww\right)}{\partial z}=\mu \left(\frac{\partial^{2}w}{\partial x^{2}}+\frac{\partial^{2}w}{\partial y^{2}}+\frac{\partial^{2}w}{\partial z^{2}}\right)-\frac{\partial p}{\partial z} \end{array},$$

The energy conservation equation is

(3) $$\frac{\partial \left(uT\right)}{\partial x}+\frac{\partial \left(vT\right)}{\partial y}+\frac{\partial \left(wT\right)}{\partial z}=\frac{\lambda }{C_{p}}\left(\frac{\partial^{2}T}{\partial x^{2}}+\frac{\partial^{2}T}{\partial y^{2}}+\frac{\partial^{2}T}{\partial z^{2}}\right),$$

where:

*u*, *v* and *w*—components of fluid velocity in *x, y* and *z* directions, respectively, m/s;

*P*—pressure, Pa;

*μ*—dynamic viscosity, Pa·s;

*T*—temperature, K;

*C_p_*—specific heat capacity at constant pressure, J/(kg·K);

*λ*—thermal conductivity, W/(m·K).

### 2.2. Mesh and Boundary Conditions

Meshing was used to mesh the above three-dimensional model, and the mesh independence was verified. The rotating part of the axial flow fan was treated with sliding mesh. The rotating velocity of the axial flow fan was 1450 r/min according to the actual operating parameters of the fan. The ambient temperature was 25 °C and the humidity was 40%. The air flow was not heated and humidified, the hole diameter of the rectifier orifice plate was 40 mm, and the hole arrangement was straight. Turbulence model using *SST k-ω.* For turbulence model, the energy equation was opened, and the convergence condition was that each residual error was less than 10^−6^. In the process of simulation, such assumptions were made: ① The air flow can flow uniformly at the inlet and outlet of the model, and the physical parameters will not change; ② the fluid is fully developed in the simulation process; ③ viscous dissipation between fluids is considered; ④ the fluid is incompressible Newtonian fluid; ⑤ ignore gravity effects.

### 2.3. Result Determination Method

The distribution of wind velocity, temperature, and humidity in the middle section of the mine roadway was basically uniform. Therefore, this paper introduced the non-uniformity judgment method to quantify the simulation and experimental results by calculating the non-uniformity of wind velocity, temperature, and humidity. Taking the wind velocity non-uniformity as an example, the wind velocity non-uniformity can quantitatively describe the flow field distribution at the test section of the device. The formula for calculating the wind velocity non-uniformity is shown in Equation (4). The calculation formula of the temperature and humidity non-uniformity refers to the calculation formula of the wind velocity non-uniformity.

(4) $$\beta_{v}=\frac{\sqrt{\frac{\sum {\left(v_{i}-\bar{v}\right)}^{2}}{n-1}}}{\bar{v}},$$

where:

$\beta_{v}$—non-uniformity of wind velocity;

$v_{i}$—wind velocity at any measurement point, m/s;

$\bar{v}$—average wind velocity, m/s;

n—number of measuring points.

When the non-uniformity does not exceed 25%, it can be considered that the flow field inside the device is uniform. Therefore, the non-uniformity of wind velocity, temperature, and humidity of the device shall be less than 25%. Furthermore, the measuring points were located on monitoring surfaces A and B, and took 16 uniformly distributed measuring points, respectively.

## 3. Results and Discussion

### 3.1. Fan Selection

The mine roadway’s main inlet and return air velocities must not exceed 8 m/s in accordance with mine safety regulations. Therefore, the device’s wind velocity must not be less than 8 m/s when the inlet fan is operating. The test pipe section has a side length of 500 mm square pipe. The air volume calculation formula says that the required air volume *Q* is where, in ideal conditions:

(5) $$Q=S\cdot v=0.5\times 0.5\times 8=2{\;m}^{3}/s=7200\;m^{3}/h,$$

*Q*—air volume, m^3^/h;

*S*—sectional area of air duct, m^2^;

*v*—wind velocity, m/s.

Under ideal circumstances, it is necessary to use the safety factor to adjust the air volume so that the fan has a sufficient supply of air. The safety factor, which is used frequently in fan selection and air volume design, is an increase of five to twenty percent based on the ideal air volume. The value of the safety factor should not be too high in order to cut down on the amount of money spent on equipment and running it. The safety factor of 1.15 was used in this paper to calculate the corrected air supply, which was:

(6) $$Q^{\prime }=Q\cdot \gamma =1.15\times 7200=8280\;m^{3}/h,$$

$Q^{\prime }$—actual air supply, m^3^/h;

*Q*—ideal air supply, m^3^/h;

γ—safety factor.

The corrected air supply volume will be used to select the fan. The type A axial flow fan can theoretically satisfy the demand for air with a volume of 10,000 m^3^/h. However, the simulation analysis revealed that the wind velocity was only 6.5 m/s after employing the type A axial flow fan. This is mostly because of the fluid’s general viscosity, the loss of vortex caused by boundary layer separation, and the loss of friction between the internal components of the fan and the air flow. Additionally, there will be some space between the fan’s static components and its rotating parts. The fan will develop a high-pressure and a low-pressure region as the impeller rotates. Leakage loss will result as a result of the air flowing through these gaps from the high pressure region to the low pressure region. The test device also has a rectifier orifice plate, a humidifying pipe section, and a heating pipe section, all of which make the air resistance when passing through the device too high and prevent the wind velocity from reaching 8 m/s.

**Figure 2:**
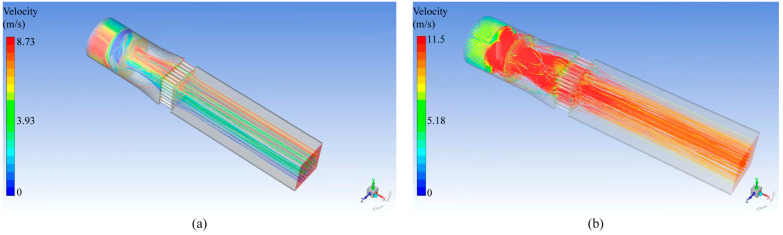
Distribution diagram of wind velocity inside the device. (**a**) Wind velocity distribution with type A; (**b**) Wind velocity distribution with type B.

According to the simulation analysis, the pipeline’s air velocity requirements call for an type B axial flow fan with a volume of 21,500 m^3^/h. As depicted in Figure 2b, the sectional wind velocity of the device can reach 8.5 m/s when the type B axial flow fan is utilized. The test pipe section’s relatively stable flow field indicates that the rectifier orifice can improve air flow uniformity. Additionally, the device’s center has a significantly higher air velocity than its near-wall counterpart. When air flows close to a wall, it has viscosity and is influenced by resistance, which reduces wind velocity. This is the primary cause of this phenomenon.

Table 1 shows the wind’s velocity and non-uniformity values of each monitoring point on monitoring surface A and B after the test device is equipped with a type B fan. According to the data in Table 1, the non-uniformity on monitoring surface A and B were 9.54% and 2.89%, respectively, both of which are less than 25%. It demonstrates that this structure’s wind velocity distribution and fan selection were reasonable.

### 3.2. Study on Wind Velocity, Temperature and Humidity

The selection of a fan may be sufficient to provide the device with sufficient wind velocity; however, it is necessary to investigate the distribution of the test device’s temperature and humidity. Miners’ primary location for carrying out production tasks is the stope. In the stope, it is necessary to measure the temperature, humidity, and wind velocity. As a result, the simulating test device must be able to completely replicate the parameters of the stope environment. The mine safety regulations stipulate that the wind velocity in the stope shall not exceed 4 m/s and the wet-bulb temperature shall not exceed 30 °C. The environment in the downhole is humid, frequently exceeding 90%. To ensure that the mine stope’s environmental parameters can be replicated, the device needs to be able to achieve a heating temperature above 35 °C and a humidity above 95% at a wind velocity of 4 m/s.

**Figure 3:**
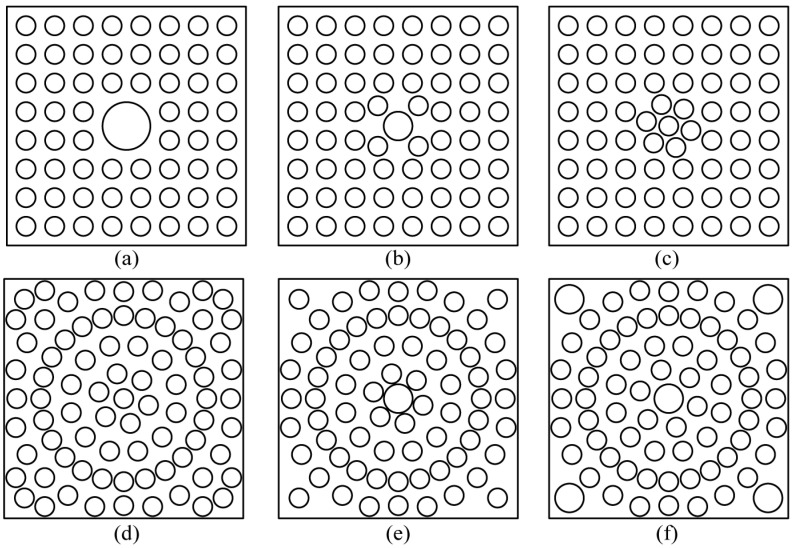
Heating tube and rectifier orifice plates with different structures and parameters. (**a**) Rectangular arrangement, center aperture is 80 mm, corner aperture is 60 mm, and other apertures are 40 mm; (**b**) rectangular arrangement, center aperture is 60 mm, corner aperture is 40 mm, and other apertures are 40 mm; (**c**) rectangular arrangement, center aperture is 40 mm, corner aperture is 40 mm, and other apertures are 40 mm; (**d**) circular arrangement, center aperture is 40 mm, corner aperture is 40 mm, and other apertures are 40 mm; (**e**) circular arrangement, center aperture is 60 mm, corner aperture is 40 mm, and other apertures are 40 mm; (**f**) circular arrangement, center aperture is 60 mm, corner aperture is 60 mm, and other apertures are 40 mm.

This method effectively avoids the non-uniform distribution of air flow caused by the phenomenon of cylindrical flow around the cylinder heating tube (Figure S1), due to the innovative device that was proposed to combine the heating tube with the rectifier hole plate. However, the optimization design of the rectifier orifice plate is subject to more stringent requirements. Because the axial fan’s center axis has a low wind velocity, the rectifier hole plate’s center position requires more air flow per unit time. The center can have either a single large hole or a dense small hole. The air flowing through a single large hole is less heated and allows for more water mist to pass through per unit time. The spray’s velocity per unit time is slowed by the dense pores’ excessively high temperature in the airflow’s center. Therefore, it is necessary to design the center structure of the rectifier hole plate in a reasonable manner so that the non-uniformity of wind velocity, temperature, and humidity does not exceed 25%. The most common approaches for optimizing the structure of the rectifier orifice design are as follows: either in a straight arrangement or a circular arrangement, alter the center aperture. There are six distinct types of rectifier orifice models constructed. Figure 3 depicts the structures and specific parameters, and numerical simulation analysis is carried out.

**Figure 4:**
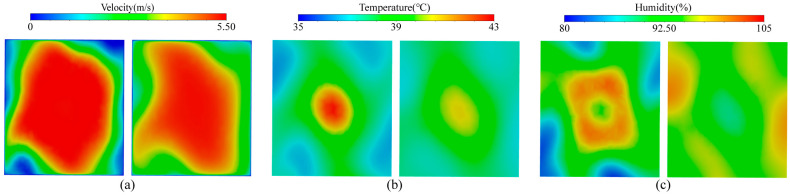
Cloud picture of wind velocity, temperature, and humidity in monitoring surfaces A (left) and B (right). (**a**) Wind velocity cloud picture; (**b**) temperature cloud picture; (**c**) humidity cloud picture.

Utilizing the breeze velocity, temperature, and mugginess non-uniformity as the assessment file, the impact of various designs of the rectifier hole plates on the lopsidedness were examined. The outcomes show that when the (f) structure with roundabout game plan, focus gap of 60 mm, corner opening of 60 mm, and other gap of 40 mm is embraced, the temperature and mugginess inside the test gadget are equally circulated. Figure 4 shows the gadget under the state of (f) rectifier hole plate, the circulation of wind velocity, temperature and dampness at two observing surfaces A and B.

Obtained by reading section data:

Figure 4a is the wind velocity distribution cloud picture. It can be seen that monitoring surface A and monitoring surface B both had high wind velocity in the center and low wind velocity at the edge. The average wind velocity on the monitoring surfaces was 4.83 m/s and 4.82 m/s, respectively. However, the wind velocity distribution of monitoring surface B was more uniform, and its non-uniformity was only 14.17%. Figure 4b’s temperature cloud picture shows that the temperature distribution of the two monitoring surfaces was high in the central area and low in the corner area, and decreased from the center to the surrounding area. The average temperatures on the monitoring surfaces were 38.19 °C and 38.23 °C, respectively. However, the temperature distribution of monitoring surface B was more uniform, and its non-uniformity was only 2.22%. As can be seen from the humidity cloud picture in Figure 4c, the humidity in the central area of monitoring surface A was higher than that in the corner, while that in the central area of monitoring surface B was lower than that in the corner area. In addition, the humidity in the central area of monitoring surface A was higher than that of monitoring surface B, while the humidity in the corner area was lower than that of monitoring surface B. The average humidity on the monitoring surfaces were 93.46% and 95.09%, respectively. However, the humidity distribution of monitoring surface B was more uniform, and its non-uniformity was only 2.40%.

Based on the analysis of Figure 4 and Table 2, it can be seen that the center of the rectifier orifice plate with structure (f) not only had a single large hole, but also a cluster of small holes. Therefore, the central part of the wind resistance was less and the wind velocity was higher. The air flow in the center was heated better by the dense pores, carrying heat as it flowed and causing it to accumulate. Because there was still a partial gap between the heating tube and the corner, there was a “heating blind area”, so the low temperature area was mainly concentrated near the corner. Due to the higher temperature of the central air flow, heat will flow from the high temperature region to the low temperature region, resulting in a lower temperature gradient. Similarly, a large amount of water mist flowed out rapidly with high wind velocity in the center, so the humidity in the center of monitoring surface A was greater than that at the edge. However, with the movement of air flow, water mist diffused to the edge during the movement of air flow, so the humidity in the center of monitoring surface B was lower than that at the edge.

## 4. Experimental Verification

**Figure 5:**
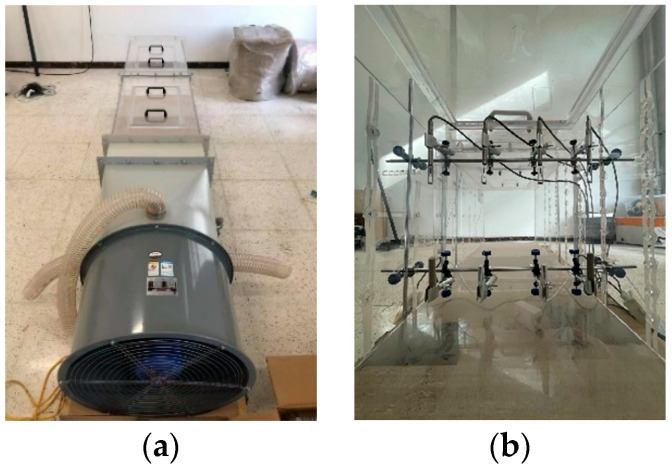
Device picture and sensor layout. (**a**) Real picture of test device; (**b**) layout of sensors inside the device.

According to the results of fan selection and rectifier orifice plate optimization, a simulation testing device of mine roadway with adjustable wind velocity, temperature. And humidity was constructed. The model was based on the similarity theory [20,21], and satisfied the geometric similarity and dynamic similarity (Equations (S1)–(S4)). The selected fan model was type B. The structural parameters of the rectifier hole plate were circular arrangement, the center aperture was 60 mm, the side aperture was 60 mm, and the other aperture was 40 mm. The heater power was 11,000 W/m^2^, and the flow rate of the water mist generator was 1.8 m/s. In addition, wind velocity sensors, temperature sensors, and humidity sensors were used for real-time monitoring of environmental parameters inside the device. In order to ensure that the temperature and humidity sensors could work normally in the simulated high temperature and humidity environment, the epitaxial waterproof highly sensitive temperature and humidity probe was selected. This probe can work stably at the temperature of 20~50 °C and the humidity of 20~100%. There were two sets of wind velocity, temperature and humidity sensors, which were located in the center of the two monitoring pipe sections (monitoring surface A and B). The arrangement of the device and sensors are shown in Figure 5.

The device is tested after the device runs stably (Figure S2), and record the wind velocity, temperature, and humidity data of monitoring surface A and B, respectively (The detailed parameters of the instrument used for the test are shown in Table S1 and Figure S3). According to Equation (4), the non-uniformity distribution of wind velocity, temperature, and humidity on each monitoring surface was solved. The test results were shown in Table 3.

As can be seen from Table 3, the average wind velocity of the two test sections of the designed and developed mine roadway simulating test device with adjustable wind velocity, temperature, and humidity were 4.70 m/s and 4.37 m/s, respectively. Additionally, the wind velocity non-uniformity were 9.54% and 2.89%, respectively. The average temperatures were 37.2 °C and 37.7 °C, and the temperature non-uniformities were 6.51% and 1.34%, respectively. The average humidity was 92% and 95%, and the humidity non-uniformity was 4.65% and 2.23%, respectively. It shows that the distribution of wind velocity, temperature, and humidity inside the device was more uniform, and all the performance could reach the design index.

## 5. Conclusions

In this study, CFD technology was combined with an experimental design. We designed and constructed a mine roadway simulating test device with adjustable wind velocity, temperature, and humidity. The device could simultaneously control wind velocity, temperature, and humidity parameters to fully simulate the tunnel environment. The results were as follows:

(1) Through air volume calculation and simulation analysis, it was concluded that axial flow fan with air supply of 21,500 m^3^/h was used in the test device. The wind velocity inside the device could reach 8.5 m/s. At this time, the minimum wind velocity non-uniformity was 2.30%.

(2) By changing the arrangement of the rectifier orifice plate, center aperture, corner aperture, and other parameters, the structure of the rectifier orifice plate was optimized. It is concluded that when the pore arrangement mode of the rectifier orifice plate was circular arrangement, the center aperture was 60 mm, the corner aperture was 60 mm, and the other aperture was 40 mm, the wind velocity inside the device could reach 4.83 m/s, the temperature could reach 38.23 °C, and the humidity could reach 95.09%. In addition, the distribution of wind velocity, temperature, and humidity in the device was relatively uniform. The lowest non-uniformity of wind velocity was 14.17%, the lowest non-uniformity of temperature was 2.22% and the lowest non-uniformity of humidity was 2.40%.

(3) According to the results of fan selection and rectifier orifice plate optimization, a simulation testing device of mine roadway with adjustable wind velocity, temperature, and humidity was constructed. The average wind velocity of the device was 4.37 m/s, and the lowest wind velocity non-uniformity was 2.89%. The average temperature was 37.7 °C, and the lowest temperature non-uniformity was 1.34%. The average humidity was 95% and the lowest humidity non-uniformity was 2.23%.

The research results showed that the device can fully simulate and reproduce the wind velocity, temperature, and humidity environment of the mine roadway. It can provide a scientific and reasonable test platform for the design and development of high-precision wind velocity sensor. It is a fundamental device for improving the wind velocity monitoring technology in the mine roadway and plays an important role in ensuring the safe production of the mine.

## Figures and Tables

**Table 1:** Average wind velocity and non-uniformity of wind velocity at each point on the monitoring surface.

	Monitoring Surface A				Monitoring Surface B			
Wind velocity (m/s)	7.89	8.15	7.95	8.25	8.44	8.55	8.44	7.88
	8.62	9.88	9.62	8.40	8.70	8.77	8.67	8.20
	8.40	9.73	10.07	8.55	8.60	8.75	8.86	8.36
	7.79	8.54	8.59	8.29	8.34	8.41	8.23	7.63
	Average wind velocity	8.58	Wind velocity non-uniformity	9.33%	Average wind velocity	8.52	Wind velocit non-uniformity	2.30%

**Table 2:** Average values and non-uniformity of indicators on the monitoring surfaces.

Monitoring Surface	Average Wind Velocity (m/s)	Wind Velocity Non-Uniformity (%)	Average Temperature (°C)	Temperature Non-Uniformity (%)	Average Humidity (%)	Humidity Non-Uniformity (%)
A	4.83	15.08	38.19	2.43	93.46	4.58
B	4.82	14.17	38.23	2.22	95.09	2.40

**Table 3:** Experimental data of wind velocity, temperature, and humidity of monitoring surface A and B.

	Monitoring Surface A				Monitoring Surface B			
Wind velocity (m/s)	4.11	4.98	4.42	4.23	4.30	4.61	4.44	4.25
	4.39	5.42	5.33	4.72	4.17	4.66	4.39	4.32
	4.53	5.40	5.35	4.61	4.36	4.37	4.41	4.43
	4.32	4.42	4.59	4.39	4.24	4.39	4.40	4.28
	Average wind velocity	4.70	Wind velocity non-uniformity	9.54%	Average wind velocity	4.37	Wind velocity non-uniformity	2.89%
Temperature (°C)	34.7	38.5	36.4	34.9	37.2	37.4	38.0	36.9
	35.6	41.3	40.8	36.7	38.2	38.6	38.3	37.4
	36.2	40.9	40.1	35.3	37.8	38.5	37.6	37.3
	34.6	36.7	37.9	34.5	37.3	37.6	37.2	37.5
	Average temperature	37.2	Temperature non-uniformity	6.51%	Average temperature	37.7	Temperature non-uniformity	1.34%
Humidity (%)	91	89	92	88	97	95	96	95
	90	98	100	91	99	94	92	96
	88	96	97	90	98	93	91	97
	84	92	90	90	96	97	95	96
	Average humidity	92	Humidity non-uniformity	4.56%	Average humidity	95	Humidity non-uniformity	2.23%

## Data Availability

The data presented in this study are available on request from the corresponding author.

## Supplementary Materials

The following supporting information can be downloaded at: https://www.mdpi.com/article/10.3390/ijerph20054057/s1, Figure S1: The phenomenon of cylindrical flow around the cylinder heating tube; Figure S2: Wind velocity stability test (from 0.5 m/s to 8 m/s). Figure S3: Test instrument. (a) Multichannel Anemomaster; (b) Wind Velocity Probe; (c) Temperature and Humidity Recorder; (d) Outward extended high-precision waterproof temperature and humidity probe. Table S1: Test instrument parameters and models.
