# Supplementary material for: Simulation Experiment Research of Mine Roadway Simulating Test Device with Adjustable Wind Velocity and Temperature and Humidity
Source: Int J Environ Res Public Health. 2023 Feb 24;20(5):4057. doi: 10.3390/ijerph20054057 (PMC10001832; doi:10.3390/ijerph20054057)
Supplement: Supplementary file 1 [file ijerph-20-04057-s001.zip › ijerph-2216680-supplementary.pdf]

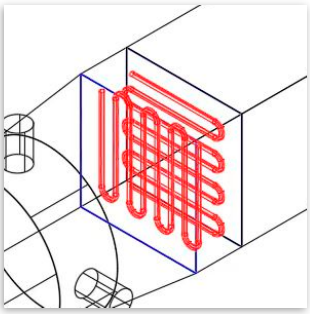

(a)

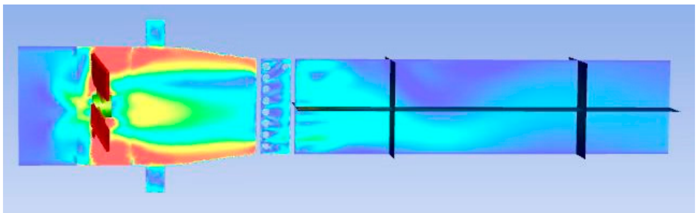

(b)

Figure S1: The phenomenon of cylindrical flow around the cylinder heating tube;

Generally, the cylindrical heating tube in Figure S1(a) is used for heating, but the cylindrical heating tube will cause the phenomenon of circular flow around the cylinder in Figure S2(b), resulting in the reduction of air flow uniformity.

The application of similarity theory in this study.

With regard to geometric similarity, the actual source of the device is a square mine roadway of approximately 2*2m, and the size is 500*500mm after being reduced by 4 times. The ratio of its actual side length L to the reduced side length a is

$$\frac{L}{a} = C_L = 4. \qquad \text{(S1)}$$

1-2. As for the dynamic similarity problem, study[1] has verified that the similarity theory can be used for the flow field analysis of the scale model of the mine roadway, and study[2] has proved that using fluent to analyze the scale model and the original scale model can obtain the same trend analysis results.

Taking Reynolds number similarity as an example, the Reynolds number formula is as follows:

$$Re = \frac{\rho v L}{\mu}, \qquad \text{(S2)}$$

assume that the actual tunnel Reynolds number is Re, the air density is ρ, the flow rate is v, the characteristic length is L, and the dynamic viscosity is μ. The Reynolds number in the device is Re', the air density is ρ', the flow rate is v', the characteristic length is a, and the dynamic viscosity is μ'. If the Reynolds number is similar, then

$$\frac{Re}{Re'} = \frac{\frac{\rho v L}{\mu}}{\frac{\rho' v' a}{\mu'}}, \qquad \text{(S3)}$$

where,

$\frac{\rho}{\rho'} = C_\rho;$

$\frac{\mu}{\mu'} = C_\mu;$

$\frac{L}{a} = C_L.$

If $Re = Re'$,then adjust

$$v' = C_\rho \times C_\mu \times C_L \times v = C \times v, \quad (S4)$$

Since the device is designed with adjustable wind velocity, if the Reynolds number is similar, the wind velocity in the device can be adjusted $v' = C \times v$. If it is assumed that the air density and dynamic viscosity do not change, then $v' \approx C_L \times v \approx 4 \times v$.

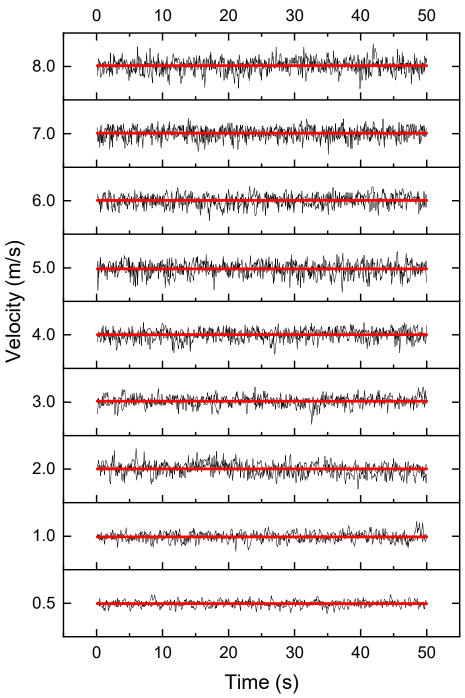

Figure S2: Wind velocity stability test (from 0.5m/s to 8m/s); the red line in the figure is the average of the measured data, and the black line is the measured data;

The wind flow stability test results show that the device can operate stably and reliably, and can be used as a test platform for wind velocity sensor.

Table S1: Test instrument parameters and models.

Category	Model	Range	Accuracy
Multichannel Anemomaster	KANOMAX 1560	Determined by probe	
Wind Velocity Probe	KANOMAX 0963-00	0.1~50m/s	0.10~4.99m/s: 0.1m/s<br>5.00~9.99m/s: 0.2m/s<br>10.0~24.9m/s: 0.5m/s<br>25.0~50.0m/s: 1.0m/s
Temperature and Humidity Recorder	RenKe COS-03	Determined by probe	
Temperature Probe	Outward extended high-precision waterproof probe	-40~+80℃	±0.1℃（25℃）
Humidity Probe	Outward extended high-precision waterproof probe	0~100%RH	±1.5%RH(60%RH,25℃)

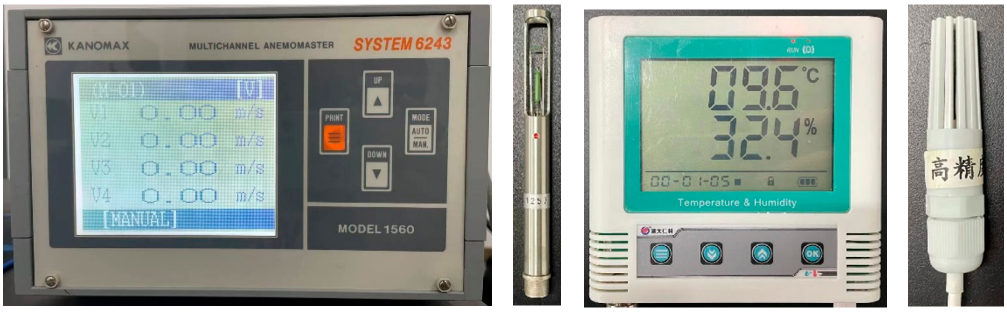

(a) (b) (c) (d)

Figure S3: Test instrument. (a)Multichannel Anemomaster; (b)Wind Velocity Probe; (c)Temperature and Humidity Recorder; (d) Outward extended high-precision waterproof temperature and humidity probe.

Table S1 and Figure S3 show the model, parameters and physical photos of the measuring instrument.
